# Falls risk perception among older adults and carers: a cross-sectional study

**DOI:** 10.1186/s12877-025-06403-9

**Published:** 2025-09-29

**Authors:** Mahbub Hasan, Bernard Walsh, Christopher Oldmeadow, Andrea Coda

**Affiliations:** 1 Royal Adelaide Hospital, Adelaide, Australia; 2 John Hunter Hospital, Newcastle, Australia; 3 Hunter Medical Research Institute (HMRI), Newcastle, NSW 2305 Australia; 4 School of Health Sciences, University of Newcastle, Callaghan, NSW 2308 Australia; 5 Equity in Health and Wellbeing Research Program, The Hunter Medical Research Institute (HMRI), Newcastle, NSW 2305 Australia

**Keywords:** Fall, Risk, Perception, Older adults, Carers

## Abstract

**Objectives:**

The main objective of the study was to explore and compare patient and carer perception of risk of falls using a concept perception of falls risk scale. We also investigated the relationship between poor perception of falls risk and potential determinants of poor perception of falls risk.

**Methods:**

A cross-sectional quantitative study was developed to capture perception of risk of falls. Informed written consents were obtained. Face to face and telephone interviews were conducted with questionnaires to explore perception of patients and carers about falls risk.

**Results:**

Participants underwent a structured interview answering survey questions related to perceptions and experiences of falls. Data was collected for 76 patients and 36 carers. 81% of the analysis population exhibited poor perception of falls risk in our concept perception of falls risk scale; this was evident among 88% of patients compared to 67% of carers. Both age and psychotropic medication were found to be potential determinants of differences in poor perception of falls risk.

**Conclusion:**

Perception of risk of fall is low among older adults and their carers. This study findings suggest participants in various settings and their carers need education about their falls risk to improve their falls risk awareness.

## Introduction

Identification of older adults at risk of falls and laying measures to prevent further falls is very important. Among the 65 years of age or above, more than every third person falls each year [1]. More than 400 potential risk factors related to falls are identified [2] namely age, history of previous falls, impaired mobility, vision and cognition, urinary incontinence, different types of comorbidities, medications and environmental factors [3]. Patient falls still occurs despite continuous implementation of fall prevention programs [3, 4]. It is also widely accepted that there are gaps in falls screening methods, programs and outcomes [3]. The falls prevention advice for others are supported by older people but not personally for themselves [5]. In general, older people have overly positive perception of their state of health, in particular their risk of falls [6, 7]. Older people minimize their susceptibilities although they understand the significance of the risk factors associated with fall [6]. Prevention of falls related health promotion messages are negatively perceived by older adults [5]. This negative perception is identified as a major factor which drives reluctance among older people’s admission to susceptibility and need for preventative behaviours to avoid falls [5, 8, 9]. It is also noted that patient perception of risks associated with fall may change their behaviours that lead to a fall [10, 11]. There is strong recommendation to include patients concerns of falling as integral part of falls risk assessment [12, 13].

Clinicians and researchers have developed falls related perception measures to evaluate various falls related constructs since the inception of falls efficacy [14]. These falls related perception instruments can be categorised into five different falls related constructs namely falls efficacy, fear of falling (FoF), balance confidence, falls behaviour and falls self-awareness [15]. Falls efficacy is defined as the perceived self-efficacy to perform activities of daily living without falling [16]. FoF is an emotional state among older adults which is the person’s anxiety towards usual walking with the perception that a fall will occur [17]. Balance confidence is defined as the confidence in one’s ability to maintain balance and remain steady [18]. Safer mobility behaviour is defined as any protective action and associated functional cognitive process used to reduce the likelihood of a fall during mobility-related activities [19]. Falls behaviour refers to actions that can either increase or decrease the risk of falls. Subjective perception of falls risk is the falls self-awareness. Falls self-awareness assess the denial and underestimation of falls risk [20].

Different falls risk measuring instruments are developed over the years to assess various falls-related constructs. Falls Efficacy Scale (FES) identifies most important activities essential for independent living that requires position change would be safe to older adults [14]. Fear of Falling Questionnaire (FFQ-R) score indicates whether a person has FoF or not [21]. Falls Behavioural (FaB) Scale is an epidemiological assessment tool [22] and its short form can be used to evaluate effectiveness of fall reduction interventions, both scales require supplementation with further items for precise application [23]. Falls Efficacy Scale International (FES-I) measures level of concerns during certain indoor and outdoor activities irrelevant of whether they perform the activity [24], development of short FES-I recommends original FES-I [25]. Activities Specific Balance Confidence (ABC) Scale assess how a person perceives their balance [18]. Self-awareness of Falls Risk Measure (SAFRM), Self-awareness of Falls in Elderly (SAFE) and Falls Risk Perception Questionnaire (FRPQ) help to measure how a person perceives their own risk of falls but requires consideration of other falls risk factors beyond self-awareness [26]. Spinal Cord Injury Falls Concern Scale (SCI-FCS) is only recommended to assess falls concern among patients with spinal cord injuries [27]. A scoping review indicated need for further evaluation of these tools, as some studies have used different falls related constructs in a manner to which they were not intended [28]. COSMIN (consensus based standards for the selection of health measurement instruments) allows systematic appraisal and selection of instruments to use in clinical practice [29]. PROMS (Patient Reported Outcome Measures) provide health care workers an opportunity to underpin patient perception in different clinical settings [15, 29]. PROMs of only FRPQ and SCI-FCS received Class A recommendations in a COSMIN systematic review [15]. FRPQ is recommended only in acute care, further studies are required to assess its use in other settings [15, 28] and SCI-FCS is recommended only for patients with spinal cord injury. There is no all-purpose measure tool for different falls construct. Instead, evaluation and measures must be tailored to each domain of falls construct that is of concern and interest.

Limited subjective perception of falls risk is a potential reason older adults taking risks leading to their falls [20, 30, 31]. The above mentioned tools supplements the fallers perception of risk of falls one way or the other, however observation, discussion and consideration of factors beyond self-awareness are required to supplement these scores. Although perception of falls risk is an important falls construct, there is lack of validated measures to assess self-awareness or perception of falls risk. The value of perception or self-reports of falls risk awareness in the context of falls is not well known [6, 10, 32, 33]. An assessment of perception of falls risk can supplement falls risk assessment, engagement and participation. This perception influences older adults’ behaviours before or after a fall and affect falls prevention strategies [33]. Here we elaborated participants self-report of falls risk awareness with a concept perception of falls risk scale utilizing a construct of subjective three item questions out of the answered interview questionnaires (Appendices).

### Aims of the study

The main objectives of the study were to:

- Explore and compare patient and carer perception of falls risk using the concept perception of falls risk scale.
- Investigate the relationship between poor perception of falls risk and potential determinants of it.

### Design

In this cross-sectional quantitative study, we captured a comprehensive event summary to a natural inquiry on perception of falls risk among older people in paucity of abundant knowledge about the concerned matter. Face to face and phone interviews were conducted to explore perception of falls among 76 patients and 36 carers. These 112 participants completed a structured interview answering survey questions related to perceptions and experiences of falls based on the Saskatoon Falls Prevention Consortium’s Falls Screening Questionnaire [34] and The Women’s Health and Aging Study [35]. A construct of three chosen survey items out of the answered interview questionnaires (Appendices) formed the concept perception of falls risk scale.

### Recruitment of participants, inclusion and exclusion criteria

Participants from only John Hunter Hospital of the Hunter New England Local Health District (HNELHD) of New South Wales, Australia were included in the present study. The study comprised of adults aged 65 and above with falls who are stable patients recovering in their acute admission, rehabilitation inpatients, community dwelling older adults of the hospital at home (HAH) programme and phone interview of recently discharged patients within last 4 weeks from the orthogeriatric and rehabilitation unit. In addition to the above, inclusion criteria of participants consisted of English speaking older adults admitted post fall without background of permanent disability or impairments, able to give consent and their available carers. Participants excluded were those unable to communicate in English, unwell, has background of permanent disability or impairments and does not have capacity to provide consent.

### Ethics

Ethics approval was obtained from the Human Research Ethics Committee (HREC) of Hunter New England Local Health District prior to embark on data collection (reference: HREC/17/HNE/514). Participation of patients and carers were voluntary. Written and verbal consent was obtained from all participants. Patient confidentiality is ensured by de-identifying all patient and carers data.

### Data collection

Data was collected from patients who were admitted post fall and their carers. Fall was explained to the participants as the subject unintentionally coming down on the floor or to a lower level not because of a major intrinsic or extrinsic event [36]. Data was collected from different clinical areas; inpatients (23.6%), rehabilitation (34.2%); HAH (27.6%) and post discharge phone interview (15.7%). Written and verbal informed consents were obtained with full disclosures from patients and carers who agreed to be interviewed and participate in this research project. Capacity to provide informed consent to participate was assessed using the University of California San Diego Brief Assessment of Capacity to Consent [37] supported by clinical judgement. When the patient or their support persons were deemed as able to consent, they were requested to complete the survey. The interviews were conducted by the patients’ bedside with the curtains screened to ensure privacy during the interview. Phone interviews were conducted ensuring privacy from isolated room in the hospital using hospital land telephone lines. Interviews were conducted in English. Patients were asked specific questions. All answers of the interviews were noted down by the interviewer. Questionnaires (Appendix A, B and C) had three sections with open ended questions, section A (Appendix A) entailed circumstances of fall, section B (Appendix B) narrated questions related to cause of recent falls, section C (Appendix C) revolved around expectations of patients on discharge. The same questions were also asked to carers (Appendix D, E and F) to explore their perceptions. Background medical history of patients and their medications were also collected to explore characteristics, associations and for comparisons. The patient records were deidentified and stored at University of Newcastle, MP Building, office: 214, Ourimbah, NSW, 2258, Australia to ensure privacy and security.

### Outcomes

Poor perception of falls risk was measured using a standard Likert Scale [38], a construct of three chosen survey items out of the answered interview questionnaires (Appendices). The questions pertained to respondent views were:

i) psychotropic medication and polypharmacy (Appendix B, E: Question 1).
ii) perceived fall re-occurrence (Appendix C, F: Question 1).
iii) inevitability of falls (Appendix B, E: Question 23).

The first question addressed if psychotropics and medications increased participated patients falls risk [39, 40]. The second question enquired whether participant is aware that person who experience one fall has twice the risk of falling again [41, 42]. People aged 65 to 84 years are more likely to experience another fall in the first 7 months after their initial fall in contrast to people aged ≥ 85 years showed a higher risk of recurrent falls after the first 7 months [43]. The last question is included in the measure of perception of falls risk as many older adults do not acknowledge their risks of fall and believe falls occur either due to bad luck or a natural consequence of ageing [5, 44–46]. Individuals with negative responses (i.e. disagree/strongly disagree) to the first two statements received a poor perception value of 1 each, indicating worse fall perception, and 0 otherwise; the scoring was reversed for the third question. Non-responses for only one question were also assumed to have poor perception. By summing the three individual responses, a total poor perception score was calculated, ranging from 0 to 3 of discrete non-negative integers; equal weighting was given for each survey measure comprising the overall outcome. If two questions were not answered, a total score was not calculated and considered missing for the participant. To investigate the aims of the study and facilitate analyses, this score was dichotomised to cases of poor perception for 2 or more items and poor perception on less than two survey measures. This was the primary outcome used in the statistical analyses for this study.

#### Predictor variables

In outlining observed perception scores and comparing levels of poor perception, respondent type was categorised as patient or carer. Patient characteristics and demographics were collected in conjunction with the fall perception survey measures. Possible categorical predictors of poor perception included gender, setting, private health insurance, type of residence, location of residence (rural/urban), psychotropic use and established dementia. Recorded continuous variables were years of age, number of falls in the past 3 months, polypharmacy and length of stay in hospital in days; if patient was at HAH programme, total number of days stayed in hospital was assumed zero.

#### Notes

Probabilities in the regression tables (predictors of the outcome) are highlighted in red for probabilities < = 0.01, yellow for probabilities > 0.01 and < = 0.05 and green for probabilities > 0.05 and < 0.2.

### General statistical methodology

Analyses were programmed using SAS V9.4 (SAS Institute, Cary, North Carolina, USA). Descriptive statistics included frequencies (and proportions) for categorical variables as well as means (SD) and medians (min, max) for continuous variables; corresponding data was approximately Normal if the mean statistic was displayed first, otherwise the distribution was skewed in nature.

For patient characteristics (*n* = 76), frequencies of categorical variables were compared between poor perception outcome categories using Chi-squared tests of independence for all variables, except when Fisher’s exact test was used (due to an expected count less than 5 in 25% or more cells). For relevant continuous variables, means were compared using a two-sample t-test with equal variances while medians were assessed using the non-parametric Wilcoxon rank-sum (Mann Whitney) method.

In terms of the complete analysis population (*n* = 112), individual total poor perception scores were summarised by respondent type. A mixed-effect logistic regression model with binomial outcome distribution and logit link function was selected to investigate potential differences in poor perception levels between patients and carers in terms of odds ratios. A random effect for dyads was also included to account for any correlation between patient and carer pairings and the Laplace method used to reduce estimate and goodness-of-fit biases.

## Results

### Descriptive statistics

81% of the analysis population (*n* = 112) exhibited poor perception for two or more items; this was evident in 88% of patients compared to 67% of carers from Table 1. This aligned with the higher mean poor perception score of 2.27 (SD 0.70) displayed by patients in comparison to 1.86 (SD 0.80) for carers; poor perception score was missing for one patient.

**Table 1:** Descriptive statistics of poor perception variable by respondent type (*n* = 112)

Characteristic	Scale/Class/Statistic	Type		Total (*N* = 112)
		Patient (*n* = 76)	Carer (*n* = 36)	
Poor Perception Score (Class)	0	1 (1.3%)	1 (2.8%)	2 (1.8%)
	1	8 (11%)	11 (31%)	19 (17%)
	2	36 (48%)	16 (44%)	52 (47%)
	3	30 (40%)	8 (22%)	38 (34%)
Poor Perception	Less Than Two Items	9 (12%)	12 (33%)	21 (19%)
	Two Or More Items	66 (88%)	24 (67%)	90 (81%)
Poor Perception Score	Mean (SD)	2.27 (0.70)	1.86 (0.80)	2.14 (0.76)
	Median (Min, Max)	2.00 (0.00, 3.00)	2.00 (0.00, 3.00)	2.00 (0.00, 3.00)

In Table 2, 51 of the total 76 patients (67%) were female; 64% of individuals with two or more items (*n* = 66) and 89% with less than two items (*n* = 9) score in the perception scale were by females. 88% of all patients lived at home while 96% inhabited in urban areas; only 6.6% had either mild cognitive impairment or mild dementia and 32% reported having private health insurance. 100.0% of patients with poor perception on less than two items were taking psychotropic medication compared to only 52% of participants who reported two or more items. Based on Chi-squared testing, this association between poor perception cases and the use of psychotropic medication was statistically significant (p-value = 0.0058, Fisher’s exact test).

**Table 2:** Descriptive statistics of patient characteristics by poor perception with relevant corresponding p-values included (*n* = 76)

Characteristic	Class/Statistic	Poor Perception		Total (*N* = 76)	*P*-value
		Less than Two Items (*n* = 9)	Two or More Items (*n* = 66)		
Gender	F	8 (89%)	42 (64%)	51 (67%)	**0.1317**
	M	1 (11%)	24 (36%)	25 (33%)	
Setting	HAH	2 (22%)	19 (29%)	21 (28%)	0.5441
	Inpatient	3 (33%)	15 (23%)	18 (24%)	
	Phone F/Up		10 (15%)	11 (14%)	
	Rehab	4 (44%)	22 (33%)	26 (34%)	
Private Health Insurance	No	7 (78%)	44 (67%)	52 (68%)	0.5026
	Yes	2 (22%)	22 (33%)	24 (32%)	
Type of Residence	Home	8 (89%)	58 (88%)	67 (88%)	0.5161
	Nursing Home	1 (11%)	3 (4.5%)	4 (5.3%)	
	Retirement Village		5 (7.6%)	5 (6.6%)	
Location of Residence	Rural		2 (3%)	3 (4%)	0.6121
	Urban	9 (100.0%)	64 (97%)	73 (96%)	
Psychotropics	No		32 (48%)	33 (43%)	**0.0058**
	Yes	9 (100.0%)	34 (52%)	43 (57%)	
Dementia	No	9 (100.0%)	61 (92%)	71 (93%)	0.3927
	Yes		5 (7.6%)	5 (6.6%)	
Age (Years)	Mean (SD)	74.44 (7.11)	82.06 (7.24)	81.30 (7.67)	**0.0041**
	Median (Min, Max)	74.00 (67.00, 90.00)	82.00 (65.00, 99.00)	81.00 (65.00, 99.00)	**0.0084**
Polypharmacy	Median (Min, Max)	12.00 (5.00, 18.00)	10.00 (2.00, 22.00)	10.00 (2.00, 22.00)	0.2147
	Mean (SD)	11.56 (4.50)	9.88 (4.34)	10.08 (4.33)	0.2823
Length of Hospital Stay (Days)	Mean (SD)	26.63 (38.83)	16.54 (25.66)	18.01 (27.25)	0.3301
	Median (Min, Max)	12.50 (0.00, 115.00)	8.00 (0.00, 141.00)	8.00 (0.00, 141.00)	0.5910
Number of Falls (Past 3 Months)	Mean (SD)	2.44 (1.59)	1.86 (1.23)	1.95 (1.27)	0.2026
	Median (Min, Max)	2.00 (1.00, 5.00)	1.00 (1.00, 4.00)	1.00 (1.00, 5.00)	0.2432

Blank cells correspond to zero frequencies for specified outcome and predictor category

Statistically significant results

In relation to individuals with two or more items of poor perception, patients that demonstrated less than two items exhibited greater mean values of days in hospital (26.63, SD 38.83 compared to 16.54, SD 25.66) and number of falls within the past three months (2.44, SD 1.59 relative to 1.86, SD 1.23). The median number of pharmaceuticals patients with less than two items of poor perception were currently taking was 12.00 (5.00, 18.00), comparatively higher than 10.00 (2.00, 22.00) in those with two or more items. None of these comparisons of predictors with poor perception were found to be significant at the 0.05 level. However, a significant difference in the mean age of patients with two or more items of poor perception (82.06, SD 7.24) compared to those with less than two (74.44, SD 7.11) was evident (*p* = 0.0041).

### Association between respondent type and poor perception level

From the results in Table 3, the odds ratio between patients and carers in terms of cases of poor perception was 4.30 (95% CI: 1.07, 17.23). Subsequently, the odds of having poor falls risk perception on 2 or more survey items if participant was a patient was 4.30 that of the odds of the same outcome for carers. As evidenced by *p* = 0.0402, respondent type was significantly associated with poor perception level.

**Table 3:** Mixed model results of dichotomized poor perception variable against respondent type using random effect of patient and carer dyads (*n* = 111)

Effect	Type	Log-Odds Ratio	Standard Error	*P*-value	95% Confidence Interval of Log-Odds Ratio	Odds Ratio	95% Confidence Interval of Odds Ratio
Type	Patient	1.46	0.68	**0.0402**	(0.07, 2.85)	4.30	(1.07, 17.23)

Location of program used to create tables: I:\PublicHealth\HBRG\CodaHasan\Programs\SAS \data_analysis.sas

## Discussion

In this study, patient views on falls were explored with respect to individual characteristics and potential risk factors. Both age and psychotropic medication were found to be potential determinants of differences in poor perception of falls risk, dichotomised by the number of survey cases.

*Comparison of Patient and Carer Perceptions:* When comparing patient and carer perceptions, the type of respondent also significantly affected the results. The only 0 score on the perception scale was by the patient whose perception score was missing. This signifies how poorly our patient cohort views their falls risk. Only one carer scored 0 in the perception scale, this finding furthers our understanding of poor falls risk perception in the community. 100% of the patients who scored at least one in the perception scale were on psychotropics, compared to 52% who scored two or more in the perception scale. Additionally, 48% patients still scored two or more in the perception scale although they were not on any psychotropics. Thus, psychotropics showed a heterogenous effect on perception in these cohort. This raises possibility of presence of a confounding variable which is unmeasured in this current analysis. Five patients in this cohort had either mild cognitive impairment or mild dementia, were able to provide consent to participate, assessed using the University of California San Diego Brief Assessment of Capacity to Consent [37]. Only one of these five patients had formal diagnosis of mild dementia. This patient had capacity to provide consent. The other four patients had cognitive scales (either Mini Mental State Examination [47] or Montreal Cognitive Assessment [48]) scores suggestive of mild cognitive impairment and had capacity to provide consent. All of them were living independently in their home or retirement village. All of them exhibited poor perception for two or more items aligned with higher poor perception of falls risk score. It is also evident that our patients cohort showed poorer perception with age increment. This finding furthers our current understanding of poor perception of risks of falls among older people [5, 33].

*Implications for Falls Prevention Strategies: *Widely used different falls constructs risk assessment tools provide a score that reflects patients propensity for falling and does not lead to reduction of falls rate [49]. Patients’ perceived risks and their actual risks differ which impacts their adherence to falls prevention strategies [10, 50]. Person who perceive a health threat may form intentions to take action and avoid harm [51]. Identifying poor perception of falls risk can facilitate person centred approach to falls management. Therefore, subjective risk perceptions assessment should be considered during designing falls prevention strategies [33]. Falls prevention is also important to reduce frailty. Older individuals who falls in the past year on an average scores 1 point higher in Edmonton Frail Scale [52].

Previously, single item question as subjective reports of memory complaints is used to assess perception [53, 54]. Similarly, two item questionnaire [33], two sets of questions [6] and three items [5] were used among community dwelling elderly individuals to assess their perception of falls risk. We developed this scale internally and undertook this pilot project to test the concept to verify its applicability. This tool has been used in clinical settings for risk screening, which informed our decision to adopt it. Three questions in our perception of falls risk scale are easy to understand risks towards a fall, derived from evidence [5, 39–46], are constant factors that are pertained to patients situation. Risk of someone who had a fall, might have another fall, constantly remains. Medication related falls risk is constantly there unless those medications are ceased. And if someone believes that falls can occur just for no reason is a perception that certainly needs attention and education regarding falls, no matter whether they already experienced a fall or not. Incorporation of perception of falls risk scale like the one we elaborated here, will likely enable health care workers with opportunities to drive improvement in reduction of falls related mortality and frailty.

### Strength

A notable strength of this study is the diversity of data collection settings, in contrasts to many currently utilized falls risk assessment tools, falls prevention programs, and perception scales that are often siloed by specific settings. This broader approach enhances the generalizability of our findings. Furthermore, the robustness of this strength can be substantiated through future psychometric validation. A scaled response by utilizing Likert Scale [38] and inclusion of three evidenced based questions [5, 39–46] in the concept perception of falls risk scale possibly increased the sensitivity of our study.

### Limitations

This study faced several limitations. Firstly, the small sample size and low individual cell counts for specific predictor categories limited the statistical power of our analyses. Additionally, the scope of the study restricted the comprehensive analysis of carer perceptions. Although multiple patients were on other falls risk increasing drugs (FRIDs), we have analysed only psychotropic medications; it will be useful to analyse the compounding effect of FRIDs on perception in future studies. Another limitation is that we have not performed clinical cognitive scales by ourselves while collecting data. Instead, we documented already completed clinical cognitive scales while participants were admitted or were in the community. It is important to acknowledge that the total perception score utilized in this study is not a validated measure, we did not conduct formal psychometric testing within our study. Further validation and appropriate testing is necessary before broader implementation.

## Conclusion

This study solely addressed perception of falls risk at this stage and it was beyond the scope of the current study to examine association between this perception and incident falls. Once validated, we plan to compare this scale with relevant tools like FRPQ and SCI-FCS. In future studies, we endeavour to evaluate drivers for changes in perception over time and whether perception can predict falls.

## Data Availability

All data and relevant materials are stored at University of Newcastle, MP Building, office: 214, Ourimbah, NSW, 2258, Australia.

## Appendix

Section A: Circumstances of your most recent fall.

We would like to know about your most recent fall. That is the fall that resulted in you.

coming to the hospital. From the following, please choose the responses that best describes that fall.

**Table:**

1	What time of the day or night did you fall?	☐ Early morning (5am − 8am) ☐ Morning (8 am − 11am) ☐ Lunch (11am − 2pm) ☐ Afternoon (2pm – 5pm) ☐ Evening (8pm − 11pm) ☐ Overnight (11pm – 5am)
2	Where did you fall?	☐ Bedroom ☐ Bathroom ☐ Kitchen ☐ Lounge room/living room ☐ Garden ☐ Garage ☐ Driveway ☐ Other (please specify e.g. shops, on footpath, on bus)
3	What were you doing before you fell?	☐ Walking ☐ Reaching (e.g. for something on a shelf) ☐ Bending over (e.g. to pick something up from the floor) ☐ Standing up (e.g. out of a chair or off a bed) ☐ Sitting down (e.g. on a chair or bed) ☐ Standing on something (e.g. ladder, chair) ☐ Getting into or out of the bath or shower ☐ Going up or down stairs ☐ Other (please specify) ☐ Don’t know
4	Were you alone when you fell?	☐Yes ☐ No
5	How long did you lay on the floor after you fell?	**________** minutes **________ **hours
6	How did you get help after you fell?	☐ My partner found me ☐ Yelled out until someone came ☐ Personal alarm system (e.g. pendant or bracelet) ☐ Call bell installed in my house ☐ Telephone ☐ Someone came to see me ☐ Other (please specify) ---------------------------------------------------------
7	How many other times have you fallen in the last 3 months?	☐ None, this was my only fall ☐ One ☐ Two ☐ More than two ☐ Don’t know
8	When was the last time you visited a podiatrist?	☐ I have never seen a podiatrist before ☐ **________ **months ☐ ________ years
9	How many times have you VISITED the emergency department in the last 12 months?	☐ None ☐ Once ☐ Twice ☐ Three or more times ☐ Unsure
10	How many times have you been ADMITTED to the hospital in the last 12 months?	☐ None ☐ Once ☐ Twice ☐ Three or more times ☐ Unsure

Section B: Causes of your most recent fall.

There may be a number of different reasons that cause people to fall. We are interested in your opinion about those reasons that may have caused your most recent fall. Please rate how much you agree or disagree with the following statements:

**Table:**

In my opinion, the most recent fall was caused by:		Strongly Agree	Agree	Disagree	Strongly Disagree
1	The number and/or side-effects of medications I take	_0_☐	_1_☐	_2_☐	_3_☐
2	Feeling unsteady and/or losing my balance	_0_☐	_1_☐	_2_☐	_3_☐
3	Feeling dizzy (e.g. standing up too quickly, vertigo)	_0_☐	_1_☐	_2_☐	_3_☐
4	Low blood pressure	_0_☐	_1_☐	_2_☐	_3_☐
5	Numbness or tingling in my legs or feet	_0_☐	_1_☐	_2_☐	_3_☐
6	The shoes I was wearing (e.g. slippers, had no grip)	_0_☐	_1_☐	_2_☐	_3_☐
7	Tripping on an uneven surface (e.g. on a mat, doorway)	_0_☐	_1_☐	_2_☐	_3_☐
8	Tripping over an object (e.g. furniture) or my pet	_0_☐	_1_☐	_2_☐	_3_☐
9	Going up or down stairs	_0_☐	_1_☐	_2_☐	_3_☐
10	Not using my walking stick, walker or wheelchair	_0_☐	_1_☐	_2_☐	_3_☐
11	Problems with my vision (e.g. blurred or loss of vision)	_0_☐	_1_☐	_2_☐	_3_☐
12	Not having anyone to help me with what I was doing at the time I fell	_0_☐	_1_☐	_2_☐	_3_☐
13	Becoming frail	_0_☐	_1_☐	_2_☐	_3_☐
14	Climbing on or off something (e.g. ladder, chair)	_0_☐	_1_☐	_2_☐	_3_☐
15	Walking in an area that was dark	_0_☐	_1_☐	_2_☐	_3_☐
16	Stiff or weak joints (e.g. due to arthritis, past injury)	_0_☐	_1_☐	_2_☐	_3_☐
17	Having alcohol some time before the fall occurred	_0_☐	_1_☐	_2_☐	_3_☐
18	Slipping on a wet surface (e.g. bathroom floor, driveway)	_0_☐	_1_☐	_2_☐	_3_☐
19	Not eating enough during the day	_0_☐	_1_☐	_2_☐	_3_☐
20	Not drinking enough water during the day	_0_☐	_1_☐	_2_☐	_3_☐
21	Not enough regular exercise	_0_☐	_1_☐	_2_☐	_3_☐
22	Exposure to chemicals (e.g. paint thinner, wood varnish)	_0_☐	_1_☐	_2_☐	_3_☐
23	Falls just happen. There’s not much you can do to prevent	_0_☐	_1_☐	_2_☐	_3_☐

Section C: What do you expect when you go home?

What are some things you are worried about once you are discharged from the hospital?

Please rate how much you agree or disagree with each of the following statements:

**Table:**

As a result of my fall and time in hospital, I am concerned that I will:		Strongly Agree	Agree	Disagree	Strongly Disagree
1	Fall and hurt myself again	_0_☐	_1_☐	_2_☐	_3_☐
2	Have to move into a residential aged care facility	_0_☐	_1_☐	_2_☐	_3_☐
3	Have to move in with my children or another family member	_0_☐	_1_☐	_2_☐	_3_☐
4	Not be able afford the care I need	_0_☐	_1_☐	_2_☐	_3_☐
5	Become a burden on my family/friends	_0_☐	_1_☐	_2_☐	_3_☐
6	Take out my frustration on people *(e.g. family*,* friends)*	_0_☐	_1_☐	_2_☐	_3_☐
7	Not be able to keep up my social activities or hobbies	_0_☐	_1_☐	_2_☐	_3_☐
8	Lose my independence and not be able to leave the house as much as I would like	_0_☐	_1_☐	_2_☐	_3_☐
9	Have to take more medications	_0_☐	_1_☐	_2_☐	_3_☐
10	Have pain that I can’t manage	_0_☐	_1_☐	_2_☐	_3_☐
11	Have to use something to help me walk (e.g. walking stick or frame)	_0_☐	_1_☐	_2_☐	_3_☐
12	Be less physically active	_0_☐	_1_☐	_2_☐	_3_☐
13	Not be able to participate in my daily activities and routines	_0_☐	_1_☐	_2_☐	_3_☐
14	Need help with personal hygiene (e.g. showering, toileting)	_0_☐	_1_☐	_2_☐	_3_☐
15	My health will get worse	_0_☐	_1_☐	_2_☐	_3_☐
16	Not know who to contact if I have a health problem	_0_☐	_1_☐	_2_☐	_3_☐
17	Have to install equipment to help prevent me from falling again (e.g. handrails, ramps, lighting)	_0_☐	_1_☐	_2_☐	_3_☐
18	Have to give away some of my belongings (e.g. furniture, mats) so my home is not as cluttered	_0_☐	_1_☐	_2_☐	_3_☐
19	No longer have control over decisions about medical care	_0_☐	_1_☐	_2_☐	_3_☐

D. Circumstances of the most recent fall.

We would like to know about the patient’s most recent fall. That is the fall that resulted in the patient coming to the hospital. From the following, please choose the responses that best describes that fall.

**Table:**

1	What time of the day or night did they fall?	☐ Early morning (5am − 8am) ☐ Morning (8 am − 11am) ☐ Lunch (11am − 2pm) ☐ Afternoon (2pm – 5pm) ☐ Evening (8pm − 11pm) ☐ Overnight (11pm – 5am)
2	Where did they fall?	☐ Bedroom ☐ Bathroom ☐ Kitchen ☐ Lounge room/living room ☐ Garden ☐ Garage ☐ Driveway ☐ Other (please specify e.g. shops, one bus, on footpath):
3	What were they doing before the fall?	☐ Walking ☐ Reaching (e.g. for something on a shelf) ☐ Bending over (e.g. to pick something up from the floor) ☐ Standing up (e.g. out of a chair or off a bed) ☐ Sitting down (e.g. on a chair or bed) ☐ Standing on something (e.g. ladder, chair) ☐ Getting into or out of the bath or shower ☐ Going up or down stairs ☐ Other (please specify) ☐ Don’t know
4	Were they alone when the fall occurred?	☐ Yes ☐ No
5	How long did they lay on the floor after the fall?	**________ **minutes ________ hours ☐ Don’t know
6	How did they get help after the fall?	☐ The partner found the patient after the fall ☐ Yelled out until someone came ☐ Personal alarm system (e.g. pendant or bracelet) ☐ Call bell installed in the house ☐ Telephone ☐ Someone came to see the patient ☐ Other (please specify) ---------------------------------------------------------
7	How many other times have they fallen in the last 3 months?	☐ None, this was the patient’s only fall ☐ One ☐ Two ☐ More than two ☐ Don’t know
8	When was the last time they visited a podiatrist?	☐ S/he has never seen a podiatrist before ☐ **________ **months ☐ ________ years
9	How many times have they VISITED the emergency department in the last 12 months?	☐ None ☐ Once ☐ Twice ☐ Three or more times ☐ Don’t know
10	How many times have they been ADMITTED to the hospital in the last 12 months?	☐ None ☐ Once ☐ Twice ☐ Three or more times ☐ Don’t know

E. Causes for the most recent fall.

There may be a number of different reasons why people fall. We are interested in your opinion about the cause(s) of the patient’s most recent fall. Please rate how much you agrees or disagree with the following statements:

**Table:**

The patient’s most recent fall was caused by:		Strongly Agree	Agree	Disagree	Strongly Disagree
1	The number and/or side-effects of medications they are taking	_0_☐	_1_☐	_2_☐	_3_☐
2	Feeling unsteady and/or losing balance	_0_☐	_1_☐	_2_☐	_3_☐
3	Feeling dizzy (e.g. standing up too quickly, vertigo)	_0_☐	_1_☐	_2_☐	_3_☐
4	Low blood pressure	_0_☐	_1_☐	_2_☐	_3_☐
5	Numbness or tingling in the legs or feet.	_0_☐	_1_☐	_2_☐	_3_☐
6	The shoes they were wearing (e.g. slippers, had no grip)	_0_☐	_1_☐	_2_☐	_3_☐
7	Tripping on an uneven surface (e.g. on a mat, doorway)	_0_☐	_1_☐	_2_☐	_3_☐
8	Tripping over an object (e.g. furniture) or the pet	_0_☐	_1_☐	_2_☐	_3_☐
9	Going up or down stairs	_0_☐	_1_☐	_2_☐	_3_☐
10	Not using their walking stick, walker or wheelchair	_0_☐	_1_☐	_2_☐	_3_☐
11	Problems with their vision (e.g. blurred or loss of vision)	_0_☐	_1_☐	_2_☐	_3_☐
12	Not having anyone to help them with what they were doing at the time of the fall	_0_☐	_1_☐	_2_☐	_3_☐
13	Becoming frail	_0_☐	_1_☐	_2_☐	_3_☐
14	Climbing on or off something (e.g. ladder, chair)	_0_☐	_1_☐	_2_☐	_3_☐
15	Walking in an area that was dark	_0_☐	_1_☐	_2_☐	_3_☐
16	Stiff or weak joints (e.g. due to arthritis, past injury).	_0_☐	_1_☐	_2_☐	_3_☐
17	Having alcohol some time before the fall occurred	_0_☐	_1_☐	_2_☐	_3_☐
18	Slipping on a wet surface (e.g. bathroom floor, driveway)	_0_☐	_1_☐	_2_☐	_3_☐
19	Not eating enough during the day	_0_☐	_1_☐	_2_☐	_3_☐
20	Not drinking enough water during the day	_0_☐	_1_☐	_2_☐	_3_☐
21	Not enough regular exercise	_0_☐	_1_☐	_2_☐	_3_☐
22	Exposure to chemicals (e.g. paint thinner, wood varnish)	_0_☐	_1_☐	_2_☐	_3_☐
23	Falls just happen. There’s not much you can do to prevent	_0_☐	_1_☐	_2_☐	_3_☐

F. Expectation for when the patient will go home.

What are some things the patient is worried about once they are discharged from the hospital?

Please rate how much you agree or disagree with each of the following statements:

**Table:**

As a result of the fall and time in hospital, the patient is concerned about:		Strongly Agree	Agree	Disagree	Strongly Disagree
1	Falling and getting hurt again	_0_☐	_1_☐	_2_☐	_3_☐
2	Having to move into a residential aged care facility	_0_☐	_1_☐	_2_☐	_3_☐
3	Having to move in with their children or another family member	_0_☐	_1_☐	_2_☐	_3_☐
4	Not being able afford the care needed	_0_☐	_1_☐	_2_☐	_3_☐
5	Becoming a burden on their family/friends	_0_☐	_1_☐	_2_☐	_3_☐
6	Taking out any frustration on other people *(e.g. family*,* friends)*	_0_☐	_1_☐	_2_☐	_3_☐
7	Not being able to keep up with social activities or hobbies	_0_☐	_1_☐	_2_☐	_3_☐
8	Losing independence and not being able to leave the house as much as they would like	_0_☐	_1_☐	_2_☐	_3_☐
9	Having to take more medications	_0_☐	_1_☐	_2_☐	_3_☐
10	Having pain that can’t be managed	_0_☐	_1_☐	_2_☐	_3_☐
11	Having to use something to help them walk (e.g. walking stick or frame)	_0_☐	_1_☐	_2_☐	_3_☐
12	Being less physically active	_0_☐	_1_☐	_2_☐	_3_☐
13	Not being able to participate in daily activities and routines	_0_☐	_1_☐	_2_☐	_3_☐
14	Needing help with personal hygiene (e.g. showering, toileting)	_0_☐	_1_☐	_2_☐	_3_☐
15	That their health will get worse	_0_☐	_1_☐	_2_☐	_3_☐
16	Not knowing who to contact if they have a health problem	_0_☐	_1_☐	_2_☐	_3_☐
17	Having to install equipment to help prevent them from falling again (e.g. handrails, ramps, lighting)	_0_☐	_1_☐	_2_☐	_3_☐
18	Having to give away some of their belongings (e.g. furniture, mats) so the home is not as cluttered	_0_☐	_1_☐	_2_☐	_3_☐
19	No longer having control over decisions about medical care	_0_☐	_1_☐	_2_☐	_3_☐
